# Synthesis and Characterizations of Novel Ca-Mg-Ti-Fe-Oxides Based Ceramic Nanocrystals and Flexible Film of Polydimethylsiloxane Composite with Improved Mechanical and Dielectric Properties for Sensors

**DOI:** 10.3390/s16030292

**Published:** 2016-02-27

**Authors:** Ashis Tripathy, Sumit Pramanik, Ayan Manna, Nabila Farhana Azrin Shah, Hanie Nadia Shasmin, Zamri Radzi, Noor Azuan Abu Osman

**Affiliations:** 1 Centre for Applied Biomechanics, Department of Biomedical Engineering, University of Malaya, Kuala Lumpur 50603, Malaysia; ayanbabu@gmail.com (A.M.); hanie_nadia@um.edu.my (H.N.S.); 2 Department of Paediatric Dentistry & Orthodontics, Faculty of Dentistry, University of Malaya, Kuala Lumpur 50603, Malaysia; nabilafarhana.shah@gmail.com (N.F.A.S.); zamrir@um.edu.my (Z.R.)

**Keywords:** polymer, nanocomposite, sintering, X-ray diffraction, flexibility, dynamic mechanical property, sensitivity

## Abstract

Armalcolite, a rare ceramic mineral and normally found in the lunar earth, was synthesized by solid-state step-sintering. The *in situ* phase-changed novel ceramic nanocrystals of Ca-Mg-Ti-Fe based oxide (CMTFO_x_), their chemical reactions and bonding with polydimethylsiloxane (PDMS) were determined by X-ray diffraction, infrared spectroscopy, and microscopy. Water absorption of all the CMTFO_x_ was high. The lower dielectric loss tangent value (0.155 at 1 MHz) was obtained for the ceramic sintered at 1050 °C (S1050) and it became lowest for the S1050/PDMS nanocomposite (0.002 at 1 MHz) film, which was made by spin coating at 3000 rpm. The excellent flexibility (static modulus ≈ 0.27 MPa and elongation > 90%), viscoelastic property (tanδ = E″/E′: 0.225) and glass transition temperature (T_g_: −58.5 °C) were obtained for S1050/PDMS film. Parallel-plate capacitive and flexible resistive humidity sensors have been developed successfully. The best sensing performance of the present S1050 (3000%) and its flexible S1050/PDMS composite film (306%) based humidity sensors was found to be at 100 Hz, better than conventional materials.

## 1. Introduction

In current technology, more than 75% of commercial miniaturized humidity sensors are based on capacitive technique [1,2,3]. Dielectric property is one of the unique properties of capacitive type sensors, and it changes with water molecules absorption in a humidity sensor. Piezoelectric ceramics are the current gold standard as multifunctional material to meet the needs of advancement in various sensor applications. Perovskite structure is one of the main structural properties of piezoelectric materials. Perovskite materials, e.g., CaCu_3_Ti_4_O_12_ (CCTO), have been explored greatly as electroceramics owing to their “giant dielectric response” [4,5,6]. Highly dielectric materials are generally suitable for capacitive type sensor devices and this capacity is also proportionally related to the capacitance, which is increased significantly with relative humidity [3]. In other words, it can also be said that the lower dissipation factor (D) or dielectric loss tangent, tanδ, (*i.e.*, ratio of energy loss per cycle, ε″ to energy storage per cycle, ε′) would benefit from improving the quality factor (Q) of any electronic device. The Q of an electronic device is a reciprocal of D. The tanδ is directly proportional to the ε″. Therefore, evaluation of tanδ is an indirect measurement of dissipation of energy under an external electric field. It includes the effects of both dielectric loss and conductivity of a material. There are also some precision capacitive methods that have shown fast response and high temperature compensation [7]. The capacitive method, which uses open capacitor as a sensing element, has fast dynamic response and also good electronic circuit with fast response [8]. The new method developed by Matko *et al.* reduces offset, temperature characteristic of main sensing element, temperature drift, and noise by switching method and it shows fast dynamic response [9]. Nevertheless, at high precision measurement, it is important to reduce any disturbing noise with good noise compensation [10]. In this instance, deconvolution method using the pseudo-stochastic excitation signals can compensate all kinds of noises and temperature drift. The titanates are the most important group for many advanced piezoelectric ceramics. They are basically perovskite M^2+^TiO_3_ in structure, and face-centered cubic (FCC) (if M = Ca, Ba, and Sr) or trigonal (if M = Fe, Co, Ni, Mn, and Mg), depending on chemical composition [11,12]. Lead (Pb) free perovskite materials have been investigated to be used in many energy storage applications [13,14]. The perovskite calcium titanate (orthorhombic or monoclinic CaTiO_3_) and geikeilite magnesium titanate (rhombohedral MgTiO_3_) are being used as attractive materials in many ceramic coating industries [15,16]. It has been found that thermal diffusivity of these materials, e.g., MgTiO_3_-CaTiO_3_, decreases with temperature but thermal conductivity increases with sintering temperature [11]. The iron oxide based composites have also been used as potential dielectric materials [17].

On the other hand, armalcolite (Fe_2_MgTi_3_O_10_) is geochemically significant, might be used as indicators of oxygen fugacity and temperature, and can be used for producing refractory ceramics and ferromagnetic materials [18]. The ilmenite (FeTiO_3_) phase is thought to be transformed into armalcolite type ((Mg,Fe^2+^)Ti_2_O_5_) after a solid state reaction with magnesium silicate (MgSiO_3_) at very high pressure (~1.4 GPa) that might have occurred by cumulate overturn process [19,20]. The phase equilibria in the MgO-FeO-Fe_2_O_3_-TiO_2_ system indicate the stability of armalcolite in the crust at 900–1200 °C [21]. Since ilmenite and armalcolite have been attempted in humidity and remote sensing applications [22,23], their derivative materials could be used as potential materials for sensors applications. However, the major limitations of ceramic materials are the undesired morphological structures and the inherent brittleness. In addition, other disadvantages of most of the ceramics are insufficient porosity and inhomogeneous distribution of pores. Especially, these two problems reduce the absorption of water or moisture and show lower hydrophilicity beside the brittleness. It significantly hinders the electrical conductivity between the microelectrodes in micro-/nano-sensors and further inhibits the sensing response to humidity sensors. To improve the flexibility of the ceramic materials, composites with polymers or elastomers perform best [3,24,25,26,27]. However, controlling the hydrophilicity is currently a great challenge to researchers for the flexible composite materials. Furthermore, flexibility of the composites was also not evaluated properly in other studies [26,27]. In this context, polydimethylsiloxane (PDMS) elastomer has shown excellent flexible property in many advanced applications, from sensors to biomedical [3,25,28,29].

Therefore, in present study, we aim to synthesize and characterize a novel submicroporous ceramic armalcolite along with a perovskite phase from some metallic (Ca, Mg, Ti, and Fe) oxides to have minimum dissipation of energy under an external electric field. The complete probable chemical reactions during mixing of oxides or sintering at high temperatures will be investigated in the present study. We also aim to prepare a flexible thin film of the armalcolite/perovskite and polydimethylsiloxane (PDMS) composite for improving the flexibility and water absorption properties along with a giant dielectric constant and improved capacitance. In this context, armalcolite, a rare earth mineral, has yet not been properly synthesized in laboratory. Thus far, armalcolite has not been investigated in potential sensing or energy storing applications. Hence, this is the first time novel synthetic armalcolite ceramics will be used as potential multifunctional materials, including charge or energy storing and electroceramic materials. In addition to this, the crystalline phases, porosity, and morphology will be controlled by an *in situ* step-sintering technique without using any further expensive methods. We also aim to develop the parallel-plate capacitive and flexible resistive humidity sensors using novel materials and their performance study at different humidity conditions.

## 2. Materials and Methods

### 2.1. Preparation of Armalcolite and Perovskite Type Oxide Ceramics

Analytical grade (99.9% pure supplied by Fisher Scientific, Selangor, Malaysia) powders such as calcium oxide (CaO), magnesium carbonate (MgCO_3_), iron oxide (Fe_2_O_3_) and titania (TiO_2_) were used as raw materials. To produce the desired perovskite and armalcolite structures, commercial CaO, MgCO_3_, Fe_2_O_3_ and TiO_2_ powders of optimized concentration 0.832, 0.877, 2.344, and 0.552 moles, respectively, were mixed in 75 mL of 70 vol % absolute ethanol. Then, Ca-Mg-Ti-Fe-based oxides (CMTFO_x_) mixed solution was mechanically wet-milled by planetary ball-mill (PM200, Retsch, Düsseldorf, Germany) at room temperature for 36 h at a constant speed of 300 rpm using alumina ball (φ 10 mm). The solid sample to balls ratio was 1:200 (*w*/*w*). The milled CMTFO_x_ powder was dried at 105 °C in a convection oven (OF-11E, Lab Companion, Seoul, Korea) for 6 h. Then, it was used to make pellets (φ 10 mm × 2.75 mm) by cold compaction pressure at 450 MPa for 2 min using uniaxial hydraulic press (GS15011, Graseby Specac, Kent, UK). The pellets were sintered by solid-state step-sintering at proper temperatures, such as 450, 650, 850, and 1050 °C, for suitable the soaking times, as illustrated in Table 1, and they are denoted by S450, S650, S850, and S1050, respectively. The *in situ* solid-state step-sintering was used to control the particle size, pore size, and porosity up to a desired range. The pellets of S1050 compound, sintered at 1050 °C, were re-crushed and dry ball-milled for 16 h at 300 rpm with sample to ball ratio of 1:100 (*w*/*w*). The probable chemical reactions during mixing at 25 °C and sintering above 450 °C or 850 °C are depicted in Reactions (i)–(iii), respectively. It indicates that during mixing at 25 °C, there was a plausible chance to produce armalcolite (Fe_2_MgTi_3_O_10_) and CaO_3_ (see Reaction (i)). After calcining over 450 °C, the produced CaO_3_ reacted with remained TiO_2_ and produced solid CaTiO_3_ (see Reaction (ii)) and carbon dioxide (CO_2_) as gas. After sintering over 850 °C, the remained Fe_2_O_3_ reacted with atmospheric oxide and converted into solid Fe_3_O_4_ and oxygen (O_2_) gas (see Reaction (iii)). Therefore, the net chemical reaction after sintering at 1050 °C is depicted in Reaction (iv).

CaO + MgCO_3_ + 2Fe_2_O_3_ + 4TiO_2_ → Fe_2_MgTi_3_O_10_ + CaCO_3_ + Fe_2_O_3_ + TiO_2_ (i)

CaCO_3_ + TiO_2_ → CaTiO_3_ + CO_2_ ↑ (ii)

3Fe_2_O_3_ + 0.5O_2_ ↑ → 2Fe_3_O_4_ + O_2_ ↑ (iii)

CaO + MgCO_3_ + 4Fe_2_O_3_ + 4TiO_2_ + 0.5O_2_ ↑ → Fe_2_MgTi_3_O_10_ + CaTiO_3_ + 2Fe_3_O_4_ + O_2_ ↑ + CO_2_ ↑ (iv)

### 2.2. Preparation of Flexible PDMS/S1050 Composite Film

At first, the sintered pellets of S1050 were re-crushed into powder form in order to incorporate in PDMS. S1050 (sintered at 1050 °C) was selected as a second phase ceramic material to make composite with PDMS owing to comprising of the desired three different phases. The flexible PDMS/S1050 composite film was explicated using the same method as described in a previous study [25]. Briefly, the PDMS/S1050 composite was first prepared by homogeneous mixing of PDMS-gel (Sylgrade184 Silicon Elastomer Base, Dow Corning, Midland, MI, USA) matrix with an optimized concentration (20 wt%) of re-crushed sintered S1050 composite powder in the ball-mill for 4 h at 300 rpm with sample to ball ratio of 1:100 (*w*/*w*). Thereafter, a referred curing agent (Dow Corning, curing agent:PDMS of 6:50 (*w*/*w*)) was added to the PDMS/S1050 mixture to render more cross-links in PDMS chains. Again, ball-milling was extended to another 8 min at 300 rpm. The uncured mixture was then spin coated on a glass petri-dish at optimized 3000 rpm for 20 s under vacuum using spin coater (650M, Laurell Technologies, North Wales, PA, USA) to make a film around 0.7 mm thick. The film was then kept under self-drying vacuum pump (PM200, Memmert, Camberley, UK) at 500 m bar for 10 min to minimize the micro-bubbles. Finally, it was properly cured in oven at 60 °C for 5 h to get the flexible PDMS/S1050 composite film.

### 2.3. Preparation of Humidity Sensors

**Figure 1:**
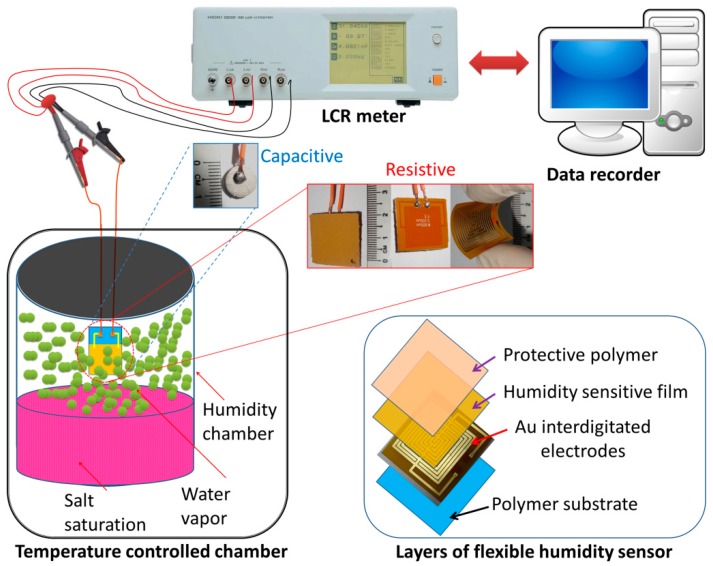
Schematic representation of capacitive- or resistive-type humidity sensors and measurement technique.

In case of capacitive-type humidity sensors, the sintered pellets were properly coated with silver paste to make a conducting surface in order to avoid the stray capacitance formation between the material and electrode. Then the silver coated pellet was dried at 250 °C for 1 h. Flexible resistive-type humidity sensors were obtained using an innovating thin film coating (TFT) technique. The mixture gel of PDMS/S1050 was deposited as a film by spin coating on custom designed interdigitated gold (Au) electrodes (800 μm in width with 200 μm spacing) of a polyimide (PI) substrate (Hansaem Digitec Co. Ltd., Incheon, South Korea) followed by heating at 90 °C for 1 h in a vacuum oven and the film thickness obtained was about 0.7 mm (see Figure 1). Finally, the humidity sensors were obtained after aging at 95% relative humidity (RH) with a voltage of 1 V, 100 Hz for 24 h to improve stability and durability.

### 2.4. Characterizations

Thermogravimetric analysis (TGA) of the unsintered ball-milled ceramic compound powder was employed by thermogravimetric analyzer (Q500, TA Instrument, New Castle, USA) in nitrogen atmosphere at a heating rate of 10 °C/min. X-ray diffraction (XRD) was conducted by X-ray diffractometer (Empyrean, PANalytical, Almelo, The Netherlands) to analyze the phase contents using CuKα radiation. Morphology of the ceramics and flexible composite were studied under field emission scanning electron microscope (FESEM) (AURIGA, Carl Zeiss, Jena, Germany). In the case of humidity measurement, it is also important to measure a high precision porosity of small specimens [30]. The pore size distribution (PSD) of unsintered and sintered (1050 °C) materials was analyzed from the inverted images of their corresponding FESEM micrographs using ImageJ software 1.46 r, as this technique is considered the best method for studying the porosity of ceramic–polymer composites [31]. Density (ρ, g/cc), open porosity (%), and absorbed water or water absoption (%) present in the porous materials were measured in water following Equations (1)–(3), respectively, using modified Archimedes’ principle explored in our previous studies [32,33]. The resolution of the weighing machine was ±0.0005 g.

(1) $$\rho \left(g/\text{cc}\right)=\frac{M_{1}}{M_{3}-M_{2}}\times \rho^{25^{o}C}$$

(2) $$P_{\text{open}}\left(\%\right)=\frac{M_{3}-M_{1}}{M_{3}-M_{2}}\times 100$$

(3) $$\text{Absorbed water}\left(\%\right)=\frac{M_{3}-M_{1}}{M_{1}}\times 100$$

where M_1_ is the initial dry mass of the samples in air, M_2_ is the mass of the specimen in distilled water, and M_3_ is the mass of the wet specimen after taking out from the water. At least five identical specimens were used to evaluate the standard deviation (SD) for each sintered sample, where $\rho^{25^{o}C}$ was water density at tested temperature 25 °C. Water contact angle (WCA) of the materials were measured using sessile contact angle meter (OCA15E, DataPhysics Instruments GmbH, Filderstadt, Germany) at room temperature (droplet size: 3 µL, dosing rate: 0.5 μL/s). The WCA was captured after 20 s for the flexible films at stable condition of the droplet, whereas WCA had to be captured instantly for all ceramic pellets since they showed super surface-hydrophilicity. Fourier transforms infrared (FTIR) spectroscopy of the samples ceramic powder sintered at 1050 °C (S1050), pristine polydimethylsiloxane (PDMS) film, and S1050/PDMS composite film was performed using attenuated total reflectance-FTIR (ATR-FTIR) spectroscope (400, Perkin Elmer, Waltham, UK) to confirm the present bonds or development of any new bonds formed between the S1050 ceramics and PDMS polymers. Flexibility of the PDMS and S1050/PDMS composite films was tested by measuring the static tensile modulus using universal testing machine (5848, InstronMicro Tester). At least three identical tensile specimens following the ASTM D 412 standard with dog-bone shape of 6.6 mm × 1.1 mm × 0.7 mm (gauge length × gauge width × thickness) were tested with a constant crosshead speed of 1 mm/min [25,34]. Force spectroscopy in tapping mode study was employed using atomic force microscope (AFM) (Nanowizard BioScience AFM, JPK Instruments, Berlin, Germany) to confirm the more precise Young’s modulus of PDMS and S1050/PDMS composite films and their corresponding topography morphology. At least five positions were selected to take more than ten times indention at each potion. Dynamic mechanical analysis (DMA) of the PDMS and S1050/PDMS composite film (size: 0.4 mm × 10 mm × 40 mm) was carried out by dynamic mechanical analyzer (RSA-G2, TA Instruments, New Castle, USA) in tensile mode at a heating rate of 10 °C/min in liquid nitrogen atmosphere in the range from −110 to −35 °C at a constant frequency of 1 Hz. Dielectric characteristics, especially storage dielectric constant (ε′) and loss tangent (tanδ = ε″/ε′), the main dielectric characteristics of materials, are very important for the electroceramic materials. Dielectric characteristics of the ceramics were measured using an impedance analyzer (4294 A, Agilent, Hyogo, Japan) over the frequency range of 10^2^–10^6^ Hz with an accuracy of ±3% done at 25 °C. The capacitive study was performed with parallel-plate capacitor structures using the impedance analyzer at 25 °C. Both surfaces of the S1050 ceramic pellet and S1050/PDMS film were coated successively with silver paste for avoiding the stray capacitance formation. Then, they were dried at 250 °C in a vacuum oven for 1 h to make them conductive with the platinum (Pt) electrodes.

The characteristics of humidity sensors were evaluated by the LCR meter. The frequency was varied from 10^2^ to 10^6^ Hz and 1 V alternating current (AC) voltage was applied for this test. The measurement was performed at 25 °C in a temperature controlled chamber (Memmet, Naluri Scientific, Schwabach, Germany) with a resolution of ±5 °C, as depicted in Figure 1. The atmospheres of different relative humidity (RH) were produced by different saturated salt solutions in several chambers. Their equilibrium states stood for 33% RH magnesium chloride (MgCl_2_), 54% RH magnesium nitrate (Mg(NO_3_)_2_), 75% RH sodium chloride (NaCl), 85% RH potassium chloride (KCl) and 95% RH potassium nitrate (KNO_3_) at 25 °C, respectively, with a resolution of about ±1% RH [35].

The response and recovery characteristic was evaluated from the time taken by the sensor to achieve ~90% of the total capacitance change in case of adsorption (humidification) and desorption (decification), respectively, of the water vapors.

## 3. Results and Discussion

### 3.1. Thermal Analysis

**Figure 2:**
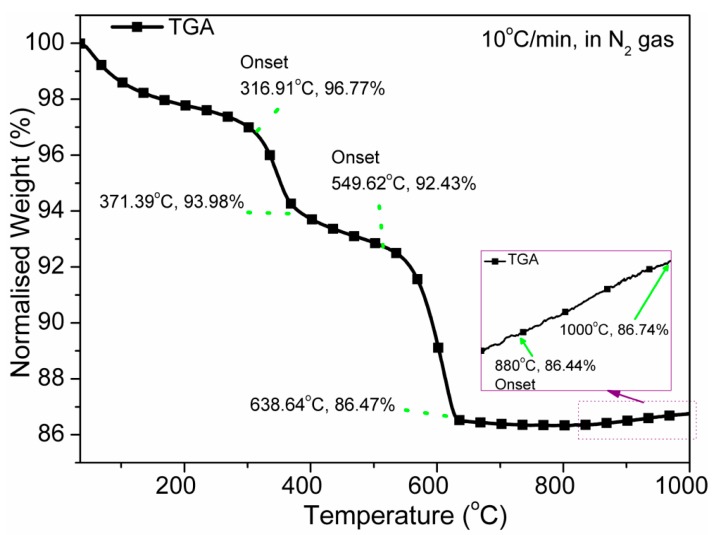
Thermogravimetric analysis (TGA) of unsintered powder from 35 to 1000 °C (ramp: 10°C/min, atmosphere: nitrogen gas). Three onset and offset points are presented. The weight-loss is obtained up to 800 °C, beyond that there is a little gradual weight gain.

After a small weight-loss (1.5 wt%) owing to removal of adsorbed water or moisture up to 120 °C, three important transitions have been found for the unsintered pellet in TGA (see Figure 2). According to TGA, the sintering temperatures were determined in order to get the different phases of the sintered materials. A larger weight change at 316.91–371.39 °C occurs due to removal of organic content such as absolute alcohol used during synthesis. The next largest weight change at 549.62–638.4 °C occurs due to decomposition of carbonate and formation of a new phase of CaTiO_3_. This result is quite similar to the CCTO materials up to this stage [36]. Further, the phase transformation was confirmed using XRD and microscopy studies. Interestingly, the weight-change of the material increases after 800 °C and indicates the *diffusion in* various other elements because of transformation Fe_3_O_4_ from Fe_2_O_3_ at high temperature. It implies that a Fe^3+^ vacancy is created at temperatures above 800 °C, which may further influence the dielectric properties of the ceramics [12,37].

### 3.2. Crystal Structures Analysis

**Figure 3:**
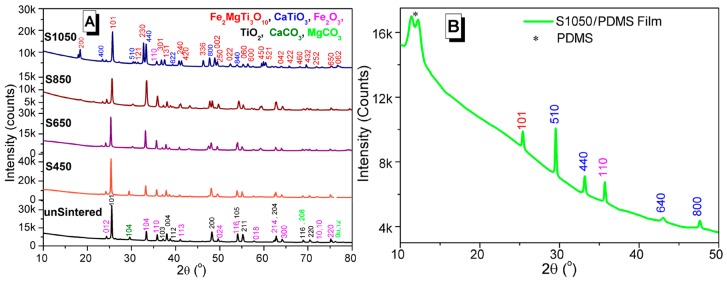
X-ray diffraction (XRD) (CuKα, λ = 1.54056 Å) patterns of the (**A**) ceramics samples unsintered (black-line); S450 (red-line); S650 (violet-line); S850 (brown-line); and S1050 (blue-line) and (**B**) S1050/polydimethylsiloxane (PDMS) composite film (light green-line). Note: the colors that denote the crystalline planes of corresponding materials are pink—Fe_2_O_3_, black—TiO_2_, green—CaCO_3_, red—Fe_2_MgTi_3_O_10_, brown—Fe_3_O_4_, and blue—CaTiO_3_.

The XRD patterns of unsintered (unSinetred: black-line) and sintered (S450: red-line, S650: violet-line, S850: brown-line, and S1050: blue-line) phase-changed ceramics are shown in Figure 3A,B for the S1050/PDMS composite (green line). In XRD pattern of unsintered ceramic, the crystal structures of all the used raw materials were noticed. The anatase-TiO_2_ (PDF:98-015-4609) shows maximum crystalline peaks along with Fe_2_O_3_ (PDF:01-084-0308), and CaCO_3_ (PDF:01-072-1650). Since no CaO peak was found in XRD pattern of unsintered material (see black-line in Figure 3A), it indicates that all CaO became CaCO_3_ after reaction with the MgCO_3_ during the wet-ball-milling. After sintering at 450 °C (see red-line in Figure 3A), it started to convert into a new phase, which partially matches with standard XRD pattern of armalcolite (Fe_2_MgTi_3_O_10_) as PDF:00-013-0353. With further temperature increases at 650 °C (see violet-line in Figure 3A) and 850 °C (see brown-line in Figure 3A), the material produces another phase of perovskite CaTiO_3_ and sharp small (400) and strong (440) planes are found, matching PDF:00-008-0092 of CaTiO_3_. The Fe_2_MgTi_3_O_10_ and CaTiO_3_ become more prominent after sintering at 1050 °C, as increasing of sharp (200) and (440) planes, respectively, and are clearly revealed in Figure 3A (see blue-line). A broad semicrystalline peak of PDMS [25] is found near 2θ = 12° along with the crystalline peaks of Fe_2_MgTi_3_O_10_ and CaTiO_3_ (see Figure 3B). It indicates that the composite is homogeneous. A small peak (110) of Fe_2_O_3_ shifted from 2θ = 35.83° to 35.70° indicates the formation of magnetic Fe_3_O_4_ that was also found in the S1050 (see blue-line in Figure 3A) as well as S1050/PDMS composite (see green-line in Figure 3B) film, which may further help in remote sensing applications [38]. The plausible chemical reactions from mixing to sintering processes have already been depicted in Reactions (i)–(iv).

### 3.3. Morphological and Surface Analyses

**Figure 4:**
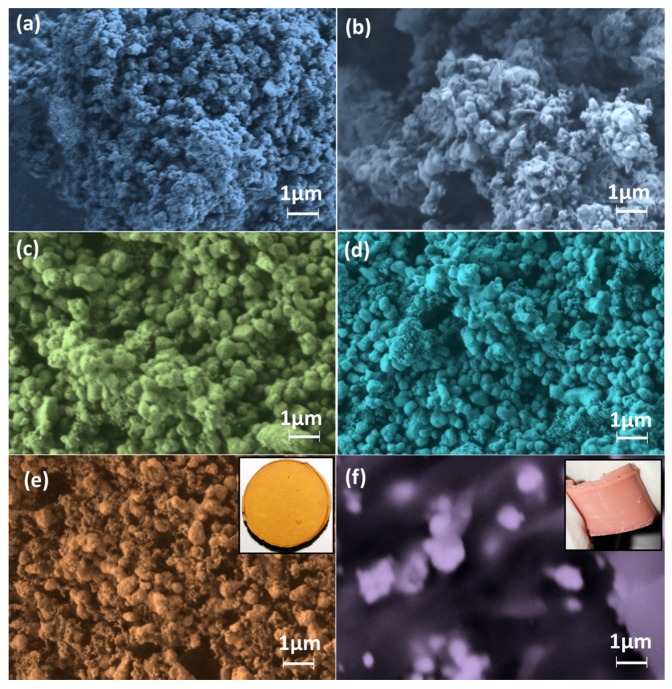
Field emission scanning electron microscope (FESEM) images of the (**a**) unsintered; (**b**) S450; (**c**) S650; (**d**) S850; and (**e**) S1050 ceramics samples; and (**f**) S1050/PDMS composite film. Note: the insets in images (**e**,**f**) are the tested pallet of S1050 and S1050/PDMS film, respectively.

A uniform submicroporous structure of the ceramic nanomaterials is revealed in the FESEM images of Figure 4. Average particle size of the unsintered ceramic (~200 nm, see Figure 4a) was found to increase at sintering temperatures 450 °C (~350 nm, see Figure 4b) and 650 °C (~750 nm, see Figure 4c). However, growth rate was controlled by changing sintering steps 850 and 1050 °C without much change in total pore distribution, where average pore sizes are 500 and 850 nm, respectively. The average particle sizes in the S850 and S1050 samples are 780 and 670 nm, respectively (see Figure 4d,e). A smaller size (typically <100 nm) of new phase of CaTiO_3_ particles has been noticed in Figure 4e. The flexible S1050/PDMS composite film shows good particle matrix bonding at the interface in Figure 4f. The digital images of the rigid S1050 pellet and flexible S1050/PDMS composite film are also depicted as inset of Figure 4e,f, respectively.

**Figure 5:**
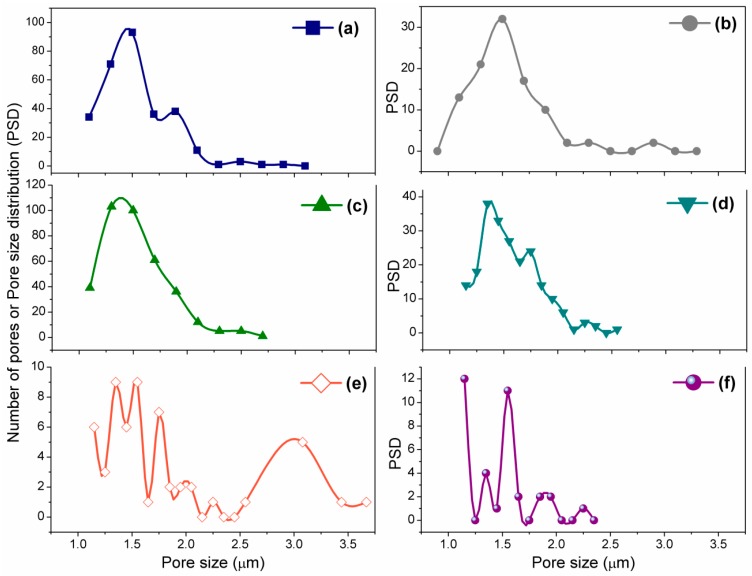
Pore size distributions (PSDs) of (**a**) unsintered; (**b**) S450; (**c**) S650; (**d**) S850; (**e**) S1050; and (**f**) S1050/PDMS were analyzed from the inverted FESEM images using ImageJ software.

Figure 5a–f depicts the calculated PSD of the unsintered and sintered (*i.e.*, S450, S650, S850 and S1050) pellets, and S1050/PDMS film using ImageJ software corresponding to their FESEM images in Figure 4a–f, respectively. It has been found that the most of the pore sizes were remained less than 1.5 μm upto sintering at 650 °C (see Figure 5a–c). The bimodal and trimodal pores were clearly revealed in the samples S850 and S1050, respectively (see Figure 5d,e) suggesting that higher sized open pores have been developed at higher sintering temperature. It is to be noted that a few large size pore also found in S450 was owing to the breaking of the loosly bonded surface of the pellets. Mainly, three modes of pore size, nearly 3 μm, 1.75 μm and less than 1.5 μm, in the S1050 indicates that three different types of claster have been developed by three different strucuted phases, armalcolite, perovskite and ferrite, which was confirmed by XRD study (see Figure 3).

**Figure 6:**
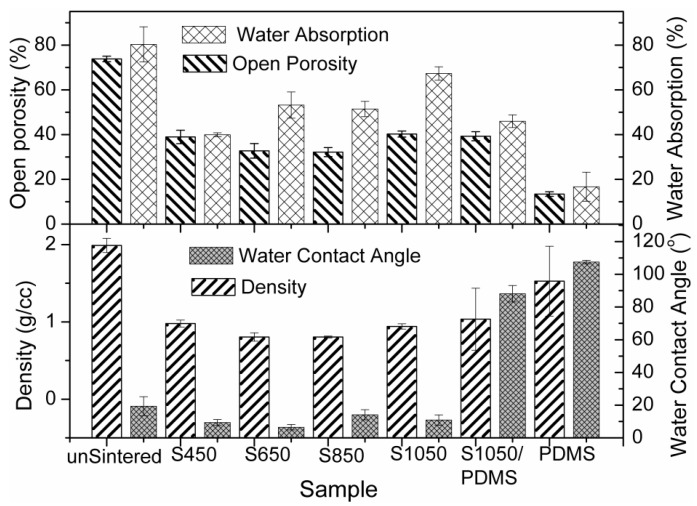
Density, open porosity, water absorption and water contact angle (WCA) of unsintered; S450, S650, S850, and S1050 ceramics samples; and S1050/PDMS composite film.

Figure 6 depicts the bulk density, openporosity, water absoption and WCA of all the materials. Bulk density was measured to evaluate the open porosity present in the materials. Lower range in density values of these pelletes compared to its comprised commercial raw materials indicates higher amount of porosity. This study also confirms that a new phase, CaTiO_3_ (see Figure 3) occurred by lattice diffusion mechanism [32,33] with increasing density (see Figure 6) by constraining the weight loss (see Figure 2) between 800 and 1050 °C. Water absorption indicates the total porosity present in the materials (see Figure 6). The higher amount of water absorption (~67%) in S1050 supports the uniform porosity that was revealed in the FESEM image (see Figure 4e). The open porosity of all these materials is lower than close porosity. Lower WCA in the ceramics strictly responsible for changing the PDMS from hydrophobic (WCA = 107°, *i.e.*, ≥90° [25]) to hydrophilic (upto WCA = 88.1°, *i.e.*, <90°) for S1050/PDMS composite film. Therefore, this flexible S1050/PDMS composite film can be used as a potential *humidity sensitive film* in many advanced remote contrlled humidity sensors [3,22,23,25].

**Figure 7:**
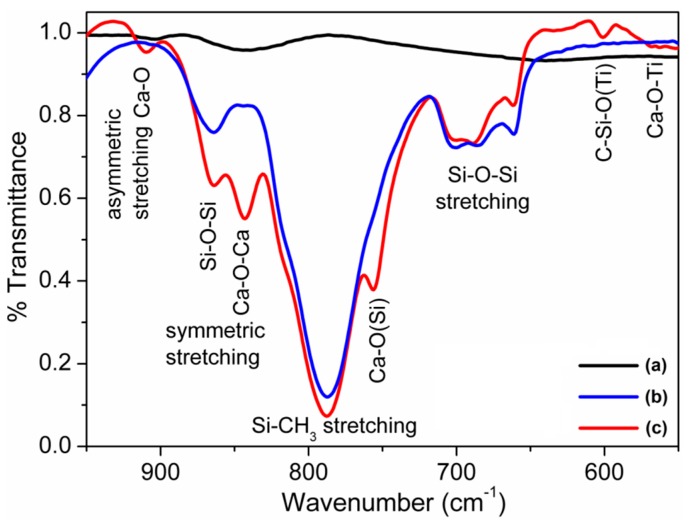
Fourier transforms infrared (FTIR) spectra of the (**a**) ceramic powder sintered at 1050 °C (S1050); (**b**) pristine PDMS film; and (**c**) S1050/PDMS composite film.

FTIR spectra depicted in Figure 7 indicate the new bond formation after developing the composite. All the FTIR peaks of pristine PDMS elastomer at 602 cm^−1^ related to Si-C stretching, 650–720 cm^−1^ attributed to Si-O-Si stretching, and 842 cm^−1^ corresponding to Si-CH_3_ stretching were also present in the S1050/PDMS composites in addition to the FTIR peaks of the S1050 ceramic at 470 cm^−1^ corresponding to Ca-O-Ti vibration from titanate and 754 cm^−1^ related to symmetric Ca-O-Ca stretching vibration [25,39]. Interestingly, the three new peaks at 600 cm^−1^ corresponding to C-Si-O from the armalcolite and PDMS, and at 754 and 910 cm^−1^ corresponding to Ca-O (Si) and asymmetric stretching of Ca-O, respectively, from titanate and PDMS reactions have been found in the S1050/PDMS composite.

### 3.4. Static Mechanical Analysis

**Figure 8:**
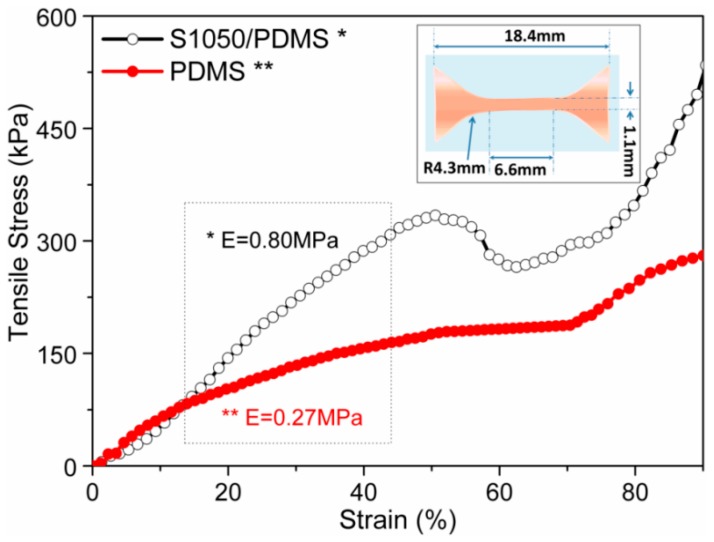
Static tensile properties of PDMS (close-symbol, red color) and S1050/PDMS composite film (open-symbol, black color). The inset is a schematic design of the film specimen according to the standard ASTM D412 method.

In order to evaluate the flexibity of the samples, S1050/PDMS composite film (open-symbol, black color) was compared with pristine PDMS film (close-symbol, red color). The Young’s modulus was measured from the selected part of the stress–strain plot of elastomers, as depicted in Figure 8. The static tensile test was performed up to 90% of the elongation. The modulus of the S1050/PDMS composite film was found to 0.80 ± 0.21 MPa, which is significantly higher than PDMS film (0.27 ± 0.08 MPa). It is worth noting that both the sample showed elongation more than 90%. This indicates that the composite S1050/PDMS composite films are as flexible as pristine PDMS film. Further, the modulus value, which is substantially lower than the other PDMS composites, indicates the higher flexibility [25].

**Figure 9:**
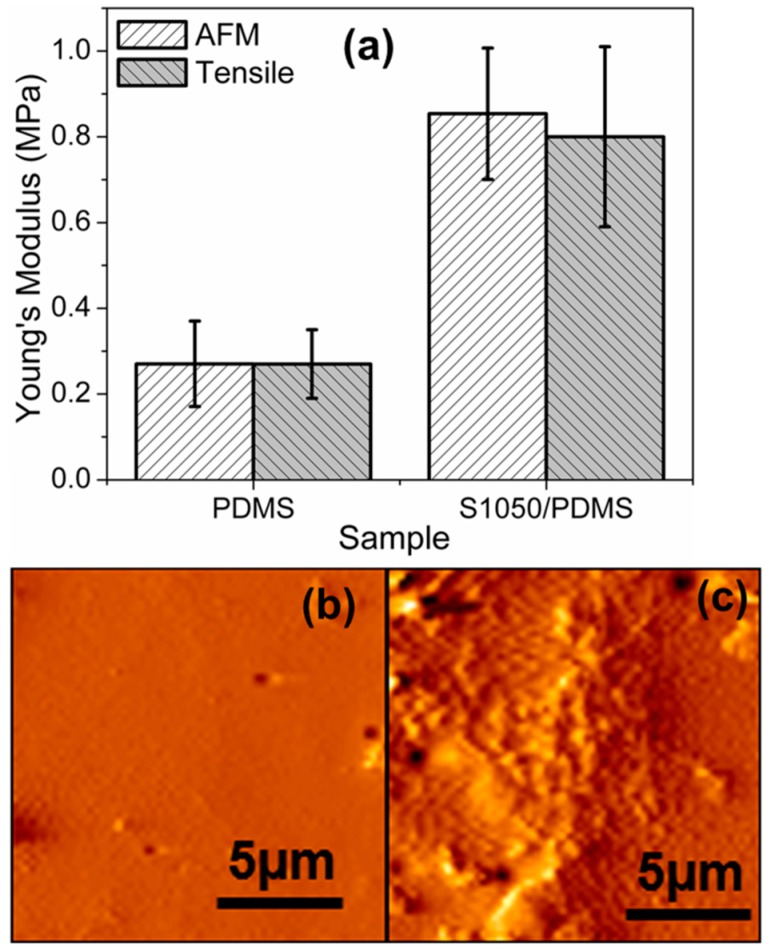
(**a**) A comparisons in Young’s modulus measured from tensile study and atomic force microscope (AFM) force spectroscopy. AFM topography of (**b**) PDMS and (**c**) S1050/PDMS composite films. Note: the Young’s moduli measured by AFM force spectroscopy for both the materials very closely matches with tensile moduli. The surface of PDMS film is smooth and surface of the S1050/PDMS composite film is rough with homogeneously dispersed particles on the top layer.

The force spectroscopy and tapping mode AFM study confirms the exact Young’s modulus and corresponding topological morphology of the PDMS and S1050/PDMS composite films. A comparison in Young’s modulus of the PDMS and S1050/PDMS composite films depicted in Figure 9a indicates the close support of the tensile moduli values for both the samples. The AFM topographs of PDMS and S1050/PDMS composite films shown in Figure 9b,c, respectively, reveal the smooth surface for PDMS film and rough surface having homogeneously dispersed particulates on the top layer for the S1050/PDMS composite film.

### 3.5. Dynamic Mechanical Analysis

**Figure 10:**
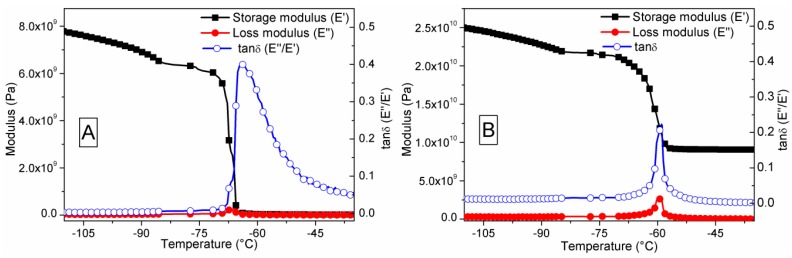
Dynamic mechanical properties in tensile mode of (**a**) PDMS and (**b**) S1050/PDMS composite film. The storage modulus value of S1050/PDMS composite increased and energy dissipation or damping factor decreased owing to formation of chemical bonds between the PDMS chains and armalcolite (Fe_2_MgTi_3_O_10_) as well as calcium titanate (CaTiO_3_) of ceramic (S1050) particles.

Figure 10 depicts the dynamic mechanical properties of the as-prepared PDMS and S1050/PDMS composite films in tensile mode. The storage and loss moduli were recorded in a cryozonic condition. The viscoelstic properties in tensile mode of PDMS and S1050/PDMS composite films are shown in Figure 10. Table 2 illustrates the glass transition temperature (T_g_), which was determined by the maximum loss in storage modulus (E′) and loss modulus (E″) or damping factor (tanδ = E″/E′), peaks occured toward higher temperature after addition of ceramic (S1050) particles in PDMS. The higher T_g_ value compared to other study indictaes the improved mechanical strength [40,41]. The higher T_g_ value obtained may be owing to the new primary or secondary bonding formations bentween the S1050 and PDMS in the composite film. The higher storage modulus value in S1050/PDMS composite film was obtained owing to addition of stiffer ceramic particles in the PDMS matrix. This result also support our static tensile test result. The value of the E′ in DMA study is higher than the Young’s modulus of static tensile mode, which was done at 25 °C, becaue of the using of cryozonic condition for DMA study. At cryozonic temperature, the mobility of PDMS polymer chains has been hindered, thus increased the stiffness or modulus of the polymeric materials. The E′ decreases significantly with increasing the temperature attributed to the transformation of glassy phase to rubbery phase. The damping factor value indicates the amount of enrgy dissipation related the molecular motion of the polymer chains during test. The lower tanδ value in S1050/PDMS composite indicates the minimum loss of energy due to applied cylcic load. The tanδ value of the PDMS based composite (S1050/PDMS) in the present study (0.225) is significantly higher than other composites of PDMS reported elsewhere [40]. It clearly indicates that the newly developed S1050/PDMS composite has higher flexibility beside having higher strength. This probably occurred due to the formation of chemical bonding between the PDMS chains and the armalcolite (Fe_2_MgTi_3_O_10_) as well as calcium titanate (CaTiO_3_) of the S1050 ceramic particles. The probable reaction is presented in Reaction (v). The new chemical band formations were also confirmed by FTIR spectra, as depicted in supplied Figure 7.

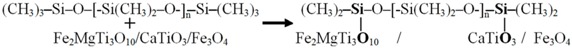
(v)

### 3.6. Dielectric Properties Studies

**Figure 11:**
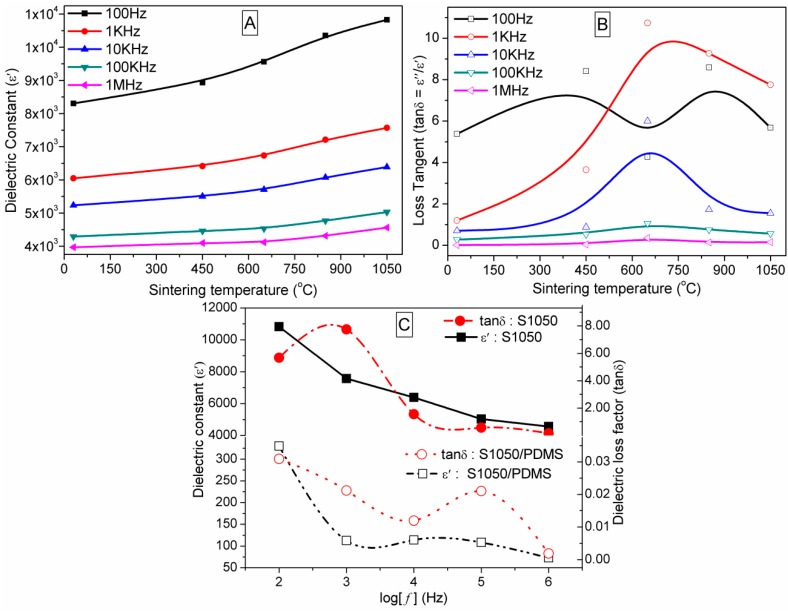
(**A**) Dielectric constant (ε′) and (**B**) dielectric loss tangent (tanδ) of unsintered (25 °C) ceramics pellet and sintered pellets, which were sintered at 450 °C (S450), 650 °C (S650), 850 °C (S850), and 1050 °C (S1050) at different frequencies 100 Hz (black), 1 kHz (red), 10 kHz (blue), 100 kHz (olive green), and 1 MHz (pink), (**C**) A typical comparisons in ε′ and tanδ of S1050 ceramic and S1050/PDMS composite film at the different log(*f*).

The effects of sintering temperature on dielectric constant (ε′) and tanδ at selected frequencies for unsintered and sintered pellets are depicted in Figure 11A,B, respectively. Figure 11C depicts a typical comparisons in ε′ and tanδ between the S1050 ceramic and S1050/PDMS composite film at different log(*f*). The ε′ gradually decreased with increasing of frequencies from 100 Hz to 1 MHz like other dielectric composites [4,6]. The ε′ gradually increases with increasing of sintering temperature from 25 to 1050 °C. The ε′ values are also significantly higher than other perovskite composites [13]. At the interface of semiconducting grain and insulating grain boundary, the charge carriers are accumulated and possessed an increase in dielectric constant [4]. It is a clear indication of formation of a new phase for all the frequencies. The new second phase was already identified as CaTiO_3_ by our XRD study and also revealed in the microstructures along with armalcolites matrix. The loss tangent (tanδ) gradually decreases with frequency change (100 Hz–1 MHz) in every unsintered or sintered material (Figure 11B). It is to be noted that loss tangent factor was lowest at 1050 °C (see Figure 11B). The higher dielectric loss in the samples at lower sintering conditions indicates the more impure or less crystalline materials were present in the materials. The lowest loss tangent (0.155 at 1 MHz) of S1050 indicates that at higher temperature the all the impurities (e.g., calcium carbonate, CaCO_3_) have been removed and the structure became more crystalline [42]. This was also confirmed by the largest transition temperature in TGA (see Figure 2) and XRD study (see Figure 3). The dielectric loss tangent factor at 1050 °C for S1050 is substantially lower than that of other ceramics [43] or silicone based composites [44] developed by other studies. This result strongly supports our previous results on dielectric loss tangent (tanδ) *vs.* sintering temperature characteristics (see Figure 11A). Therefore, the S1050 material can be used as a potential dielectric material for sensors and electronic device applications. Furthermore, Figure 11C indicates that the ε′ (*i.e.*, 72–330) of the S1050/PDMS film is considerably lower than our S1050 ceramic but significantly higher than other pure PDMS or PDMS based materials, which show generally less than 10 [45]. The excellent enhancement in ε′ has been obtained owing to the incorporation of giant dielectric material such as S1050 ceramic particles homogeneously in the PDMS matrix. In addition, the tanδ value of S1050/PDMS film is significantly lower than that of S1050 ceramic, indicating the very low loss in energy dissipation, which will be discussed later in more detail.

**Figure 12:**
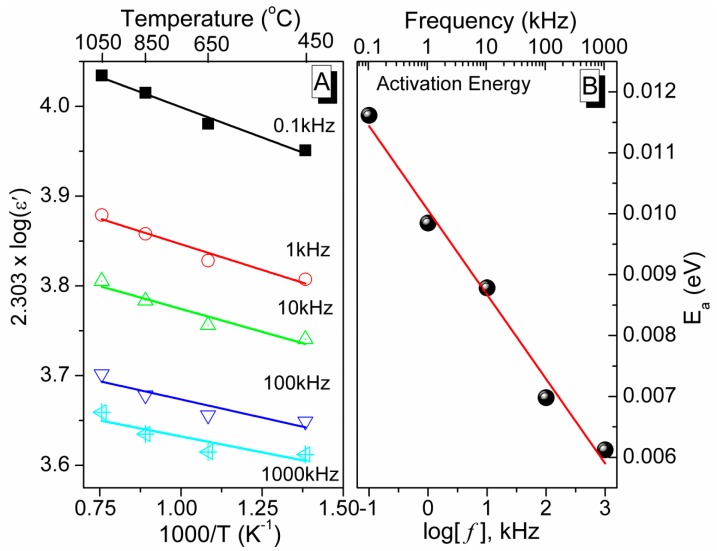
(**A**) Arrhenius plots of sintering temperature dependence dielectric constants of the sintered pellets and (**B**) activation energy (E_a_) of the sample at frequencies 0.1, 1, 10, 100, and 100 kHz. Note: the symbols represent the experimental data and lines represent the linear fitted curves.

The dielectric constant (ε′) of the present sintered ceramic materials also followed Arrhenius law, as given in Equation (4).

(4) $$\varepsilon \prime =\varepsilon_{o}\text{exp}\left[-\frac{E_{a}}{k_{B}}\cdot \frac{1}{T}\right]$$

where ε_o_ is the pre-exponential factor, T is the sintering temperature (in K) at different stable phases, E_a_ is an activation energy (in eV) and k_B_ is the Boltzmann constant (8.6173 × 10^−5^ eV/K). The obtained activation energy required to shift from one stable phase to another stable phase (as mentioned in the TGA and XRD studies) can be evaluated from the slope of the plots log(ε′) *vs.* 1/T, as depicted in Figure 12A. It has been found that the activation energy of the newly developed ceramic material decreased linearly with increasing of frequency in logarithm scale (see Figure 12B). The regression values of the Arrhenius plots in Figure 12A were found to decrease with increasing of frequency (*R*^2^ = 0.97322, 0.94001, 0.90212, 0.78117, and 0.68378 for frequency 0.1, 1, 10, 100, and 1000 kHz, respectively), indicating the ε′ is more prone to follow the Arrhenius law at lower frequencies. The regression value of the activation energy plot in Figure 12B, which is close to 1 (*R*^2^ = 0.984), indicates the following of linear change with frequency in logarithm scale.

**Figure 13:**
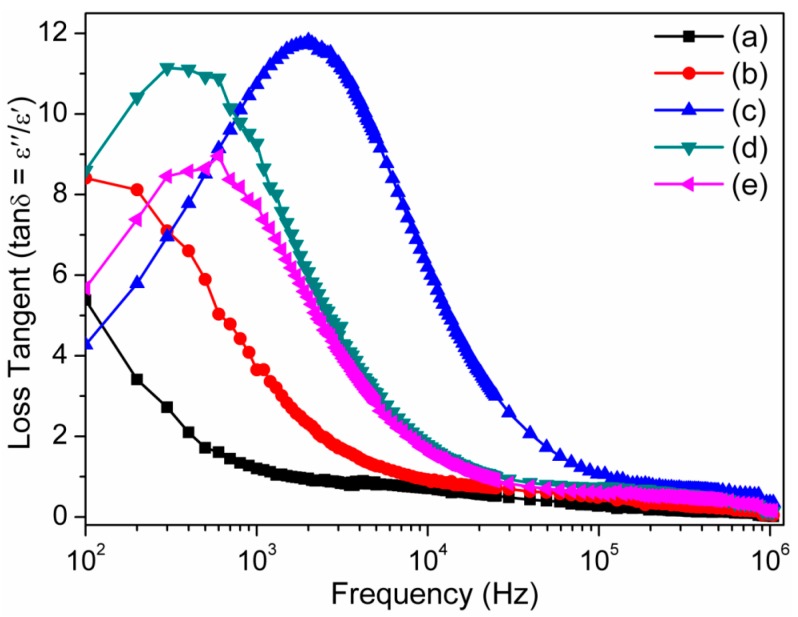
Frequency dependence dielectric loss tangent (tanδ) of the unsintered pellets (**a**) at 25 °C (black) and sintered ceramics pellets and those sintered at (**b**) 450 °C (red) (S450); (**c**) 650 °C (blue) (S650); (**d**) 850 °C (olive green) (S850); and (**e**) 1050 °C (pink) (S1050).

Figure 13 shows that the tanδ values of the sintered CMTFO_x_ ceramics at different sintering temperatures first gradually increases and then decreases with increasing of frequencies from 100–20,000 Hz and then almost constantly changes up to 1 MHz in logarithm scale. The single maxima in ceramic materials indicate that they have a relaxation [4,6]. In the sintered ceramic materials, the prominent tanδ peak at the lower frequency region up to 2000 Hz is attributed to the space charge polarization, which was developed because the two phases of grain–grain boundaries of different electrical conductivities are in contact [4]. The different phases of two distinct sizes morphology have also been found in our FESEM micrographs (see Figure 4b–e). On the other hand, the unsintered material has single morphology (see Figure 4a) and thus has shown insignificant tanδ peak. The tanδ peak shifts toward higher frequency with increasing sintering temperature, indicating more space charge polarization due to rising number of grain boundaries. An exceptional shift of tanδ peak for S650 might be due to the extremely tiny second phase particles, which were just nucleated at 650 °C. The loss tangent also lowered at higher frequencies and temperatures (850 and 1050 °C). This result clearly indicates that the higher dielectric constant (ε′) or lower dielectric loss (ε″) can be obtained for the pellets at higher sintering temperature. This result is an indication of the development of purer and more crystalline phases at higher temperatures. The dielectric constant (ε′) and tanδ obtained from the present study have been substantially improved compared to the PDMS based or other CCTO based composites reported elsewhere [4,6,46,47].

**Figure 14:**
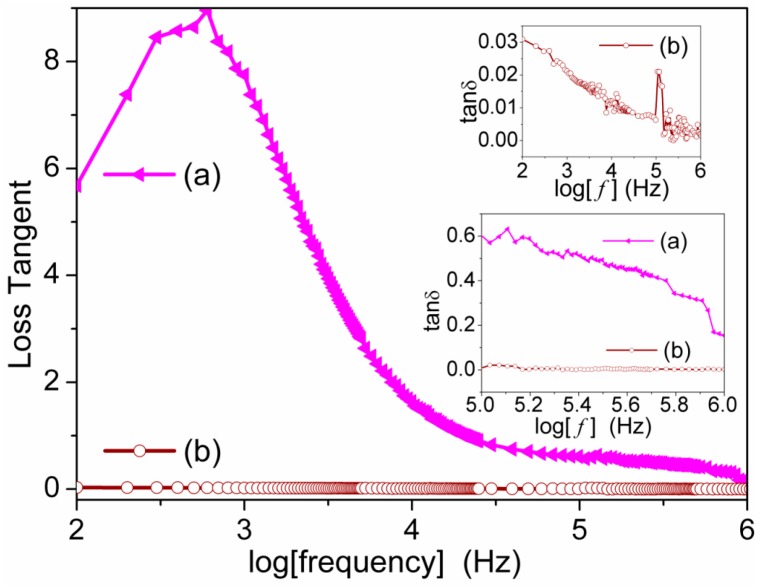
Frequency dependence dielectric loss tangent (tanδ) of (**a**) sintered pellet at 1050 °C (pink (S1050)) and (**b**) composite film (brown (S1050/PDMS)). The inset represents the precise observation at 10^5^–10^6^ Hz on the tanδ changes for ceramic pellet of S1050 and flexible film of S1050/PDMS composite.

The dielectric tangent loss of the S1050/PDMS composite is compared with the S1050 ceramic material in Figure 14. It is has been found the the dilectric tangent loss factor of the S1050/PDMS composite film (0.02–0.002) is much lower than that of S1050 ceramic pellet (5.75–0.15) in the range of 100 Hz to 1 MHz frequency region. A precise observation at a frequency ragne of 10^5^–10^6^ Hz, similar to a previous study [6], on the change in tanδ value for S1050 ceramic pellet and flexible S1050/PDMS composite film is depicted in the bottom inset of Figure 14. It indicates that the PDMS has significantly influenced the dielectric properties of ceramic and *vice-versa*. This might be because the new bonding formation between the silicon of backbone chains of PDMS with the compositions of S1050 ceramic. The newly developed bands have been found in Fourier transforms infrared (FTIR) spectra, which is depicted in supplied Figure 7. The dielectric loss tangent of the the S1050/PDMS composite film also resembled the values of PDMS [28]. The lower dielctric tangent loss is a clear indication of improvement of the quality factor of sensors or electronic devices. Hence, it evidently suggests that the sensing properties of the S1050/PDMS composite film has potential for sensor device applications. The frequency dependence of the dielectric tangent loss (tanδ) of S1050/PDMS composite at very precise observation is depicted in the top inset of Figure 14 and indicates two relaxations. The tanδ loss peak at lower frequency region nearly 100 Hz occurred due to the relaxation attributed to molecular motion in the crystalline regions of PDMS polymer chains as well as grain–grain boundary interfacial polarizations [4,6] and the second sharp peak at higher frequency region near 10^5^ Hz indicates another relaxation associated with the glass transition of PDMS polymer [6].

**Figure 15:**
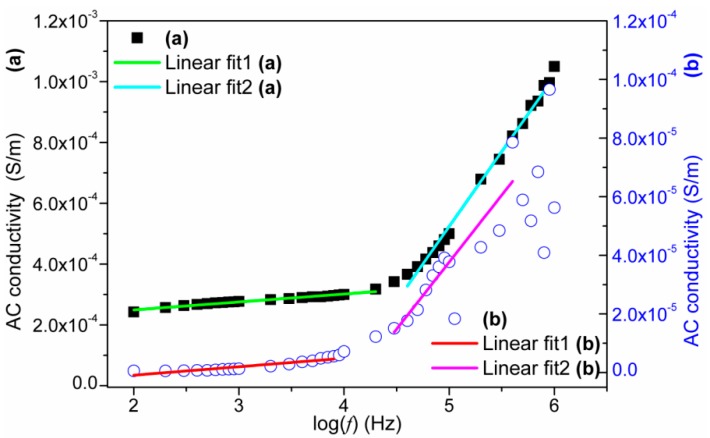
Frequency dependence AC conductivity (σ_ac_) of (**a**) sintered pellet at 1050 °C (pink (S1050)) and (**b**) composite film (brown (S1050/PDMS)). Both materials show two different slopes below and above 5 × 10^4^ Hz, indicating a transition in electrical conductivity.

A frequency dependent AC conductivity (σ_ac_) of the S1050 pellet and S1050/PDMS composite film is depicted in Figure 15. Both materials showed a slower and a faster increment below and above log(*f*) = 4.4 Hz, respectively, clearly suggesting that an insulator-semiconductor transition zone in electrical conductivity occurred near 25 × 10^3^ Hz. This phenomenon in the crystalline oxide based materials is attributed to the ionic hopping mechanism, which is highly possible in both sintered crystalline S1050 and its flexible composites film (S1050/PDMS) [48,49].

**Figure 16:**
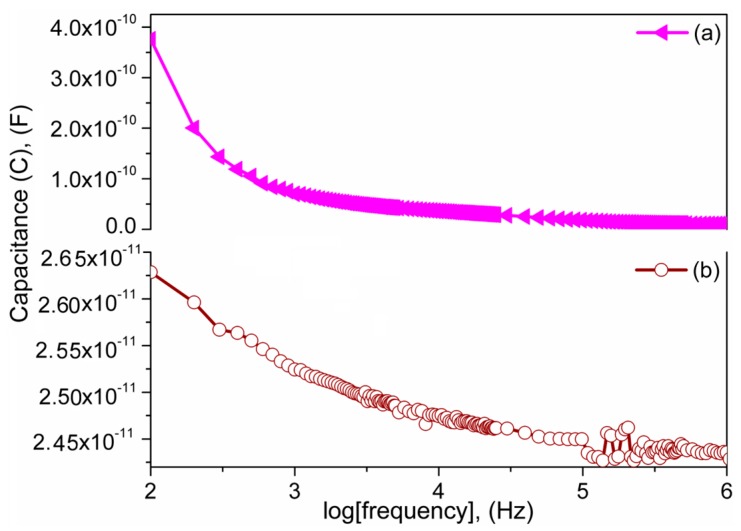
Frequency dependence capacitance of (**a**) sintered pellet at 1050 °C (pink (S1050)) and (**b**) composite film (brown (S1050/PDMS)). The S1050/PDMS composite film represents two significant peaks in between 10^5^ and 10^6^ Hz that were not shown by S1050 ceramic.

The frequency capacitance of the S1050/PDMS nanocomposite is compared with the S1050 ceramic dielctric material in Figure 16. Since dielectric constant, which is directly proportional to the capacitance value (C = ε′ε_o_A/t, where C is capacitance, ε′ is real dielectric constant, ε_o_ is the free space permittivity, A is cross-sectional area and t is the thickness of the sample), of the S1050 was significantly higher at all frequency (see Figure 11), its capacitance value would be best in comparison with unsintered or any other sintered material. Therefore, only S1050/PDMS composite could be used to develop a flexible composite film with potentially the best in performance. The capacitance values of the S1050 ceramic and S1050/PDMS composite film were in the order of 10^−10^ F and 10^−11^ F, respectively, which closely resembled those of different nanoparticles and composites [50,51]. The capacitance value of the S1050/PDMS composite film is significantly higher than the other titanate fibrous materials [51]. Therefore, the S1050/PDMS composite film is capable of storing large amounts of electrical energy, which is necessary for several electronic and power devices [27]. The capacitance values of both dilectric materials also followed a decreasing trend with frequency. This is obviously due to the loss of ionic polarizations with increasing of frequency. The grain–grain boundary interfacial polarizations and molecular motion in the crystalline regions of PDMS polymer chains increase the capacitance value of the S1050 ceramic and S1050/PDMS composite film, respectively, at lower frequencies (100–3000 HZ). At a higher frequency range, 10^5^–10^6^ Hz, the two pikes of capacitive value in S1050/PDMS composite film is attributed to the glass transitions, such as α- or β-transition, of PDMS polymer. The first peak may be related to a breakage of weak secondary bond, such as Van der Waals dipole interactions, between the ceramic particles and polymer chain and second peak may be owing to another, stronger secondary bond, such as hydrogen bonds within the PDMS polymer chains [52].

**Figure 17:**
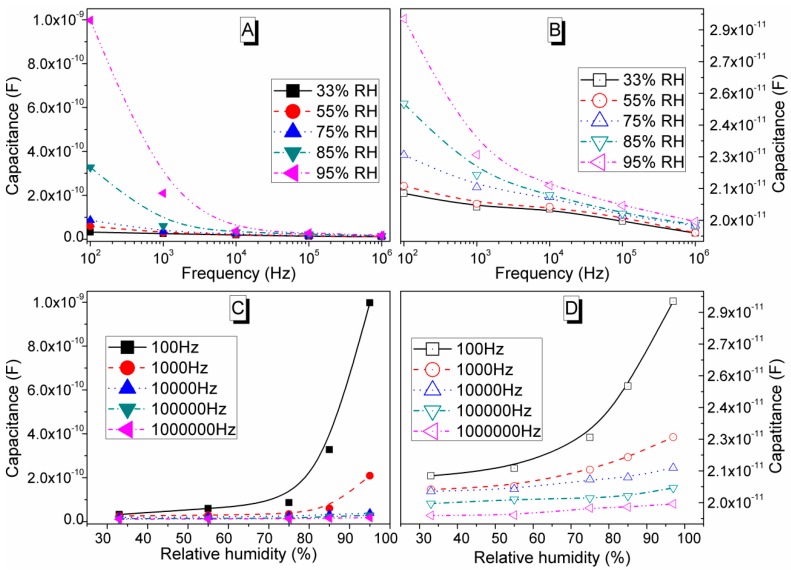
Frequency dependence capacitance of (**A**) sintered pellet at 1050 °C (S1050) (filled symbols) and (**B**) composite film (S1050/PDMS) (opened symbols) at different humidity conditions (33, 55, 75, 85, and 95 % RH) in the frequency range of 10^2^–10^5^ Hz. The capacitance response with % RH condition at different frequency points (10^2^, 10^3^, 10^4^, 10^5^ and 10^6^ Hz) for (**C**) sintered pellet at 1050 °C (S1050) (filled symbols) and (**D**) composite film (S1050/PDMS) (opened symbols).

The relationship between capacitance and frequency (10^2^–10^6^ Hz) at RH range of 33%–95% RH of the S1050 pellet and flexible S1050/PDMS nanocomposite film is depicted in Figure 17A,B, respectively. It shows that capacitance increases with increasing of % RH at low frequencies up to 10^3^ Hz. However, at higher frequencies (>10^3^ Hz), the change in capacitance becomes almost independent of the RH and the capacitance value reduced significantly. Both materials showed similar trends but the capacitance values for the porous ceramics were almost an order of magnitude higher compared to the composite film. The result is quite expected since hydrophobic PDMS polymer is used as matrix material in the S1050/PDMS nanocomposite. The S1050 ceramic played an active role in the humidity-sensing layer because of the hydrophilic characteristics of its porous surface where numerous nanopores help improve the water molecule adsorption/desorption process and enhance the sensitivity of the sensor. The capacitance-change trend of these materials indicates that the electrical field direction changes slowly at low frequency and then with the appearance of the space-charge polarization owing to adsorbed water. Higher % RH implies more adsorbed water, stronger polarization, and, thus, larger dielectric constant, impedance and capacitance. When the frequency is high, the electrical field direction changes at such a faster speed that the polarization of the water cannot catch up with it, and hence the dielectric constant becomes smaller and independent of % RH [53,54]. A similar observation was also obtained for the capacitance response with % RH condition at different frequency points (see Figure 17C,D).

To evaluate the humidity dependent capacitive characteristic of the S1050 electroceramic and humidity dependent resistive characteristic of flexible S1050/PDMS nanocomposite film, the device sensitivity (S) is calculated using Equation (5) [55] and Equation (6) [56], respectively.

(5) $$\text{Sensitivity }=\frac{C_{RH}-C_{33}}{C_{33}}\times 100\%$$

(6) $$\text{Sensitivity }=\frac{\Delta Z}{\Delta \%\mathrm{RH}}$$

where C_RH_ is the capacitance at any measured % RH condition and C_33_ is capacitance value at the initial of RH is equal to 33%, Δ% RH is the difference in % RH and ΔZ is the difference in impedance or resistance. The value of capacitance increased from 3.2183 × 10^−11^ F to 9.9741 × 10^−10^ F, with S of 3000% at the signal frequency of 100 Hz. This value is improved in comparison to the other materials (3000%, even at 85 Hz) [57]. It has been found that at test frequency 10^5^ Hz, the S comes down to 120% as the capacitance varied from 1.2832 × 10^−11^ to 2.8174 × 10^−11^ F across the RH range of 33%–95%. On the other hand, for flexible S1050/PDMS nanocomposite film, the S is found to be 306 and 1.212 kΩ/% RH at 10^2^ and 10^5^ Hz, respectively, in the range of 33%–97% RH. The value of S at 10^2^ Hz is significantly higher than other resistive type humidity sensors materials (S = 160 kΩ/% RH for BaTiO_3_) [58]. Therefore, the present electroceramic (S1050) and its flexible composite film have shown the best sensor characteristic performance at test frequency 10^2^ Hz.

The response and recovery result of our capacitive sensor showed that the response time for humidification (from 33% RH to 95% RH) was 14.5 s and recovery time for desiccation (from 95% RH to 33% RH) was 34.3 s. Therefore, the obtained result, specifically response time of the present capacitive humidity sensor is noticeably better than conventional capacitive sensors such as silicon nanowires (response time = 132 s at 11.3% RH to 93% RH) [59], anodic aluminum oxide (response time = 188 s, 30% RH to 95% RH) [60], and so on.

## 4. Conclusions

A novel sensing ceramic with a flexible composite film and suitable humidity sensor has been designed and developed in the present study. The morphology of the armalcolite and perovskite phased ceramic compounds was obtained by solid-state step-sintering technique, which was determined by TGA. The complete chemical reactions after ball-milling at 25 °C and calcining or sintering up to 1050 °C for the ceramic powders have, for the first time, been unveiled in the present study. Different phases of the sintered material have been distinctly determined by XRD technique. The new CaTiO_3_ phase of smaller nanoparticles was found to be formed by *in situ* solid-state step-sintering via lattice diffusion mechanism in the temperature range of 800–1050 °C. Most homogeneous uniform structure of porous sintered ceramic materials was found at 1050 °C. According to the higher water absorption, lower WCA, and optimum density, the S1050 was selected for making the composite film with PDMS elastomer. The flexibity of the S1050/PDMS composite film has clearly been noticed by subpressing of tesile modulus and improved elongation compared to other PDMS composites [25]. It can be noticed that both samples showed more than 90% tensile elongation. The improved viscoelastic property such as lower damping factor of the S1050/PDMS composite (tanδ = 0.225) compared to the pritine PDMS film (tanδ = 0.404) indicates higher mechanical strength. The dielectric loss tangent of the newly developed S1050/PDMS (0.02 and 0.002 at 100 Hz and 1 MHz, respectively) and S1050 ceramics (5.75 and 0.155 at 100 Hz and 1 MHz, respectively) is noticeably lower than that of the other ceramics or composites of silicone rubber reported by Tiercelin *et al.* (2006) [28]. This shows promise for improving the quality factor of sensors or electronic devices. The dielectric constant of the newly developed sintered CMTFO_x_ and S1050/PDMS composite is also significantly higher than the other recently developed nanocomposites by Nayak *et al.* (2013) [27]. This flexible “giant dielectric” material would be an ideal replacement of CCTO based brittle materials [6]. The improved capacitance values of S1050 ceramic and S1050/PDMS film can promisingly be used for capacitive type humidity sensors [2]. In case of humidity sensors, higher % RH introduced more adsorbed water molecules in both the pellet and film based humidity sensors, and thus stronger polarization, which finally improves dielectric constant, impedance and capacitance. Since the particle sizes of the ceramics, comprising armalocolite, CaTiO_3_ and Fe_3_O_4_, are in nanoscale, the thin film can be designed to be less than 1 μm, which is necessary for miniaturization of advanced electronic devices. Therefore, capacitance values of the newly developed ceramic as well as S1050/PDMS flexible composite film are very promissing, along with the “giant dielectric” constant. Hence, the S1050/PDMS composite film or membrane, having higher flexibility, hydrophobicity and improved dielectric properties, could be a potential material in capacitive and resistive-type sensors for remote and humidity sensing applications. From our present work, the best performance study of the present electroceramic (S1050) or its flexible composite film would be at test frequency 10^2^ Hz for the analysis of humidity sensor characteristics.

## Figures and Tables

**Table 1:** Schematic sintering steps of samples S450, S650, S850, and S1050.

Sample	Step-I		Step-II		Step-III		Step-IV	
	Temperature for Time	Rate (°C/min)	Temperature for Time	Rate (°C/min)	Temperature for Time	Rate (°C/min)	Temperature for Time	Rate (°C/min)
S450	450 °C/3.5 h	5						
S650	250 °C/1 h	5	650 °C/3.5 h	10				
S850	350 °C/1 h	5	550 °C/3.5 h	10	850 °C/1.3 h	10	750 °C/3 h	20
S1050	350 °C/1 h	5	550 °C/3.5 h	10	1050 °C/1.3 h	10	750 °C/3 h	20

**Table 2:** Dynamic mechanical properties of PDMS and S1050/PDMS films.

Material	Maximum E′ (Pa) at 110 °C	Temperature (°C) at Maximum Loss of E′	Temperature (°C) at E″ Peak	Temperature (°C) at tanδ Peak	tanδ Value (E″/E′)
PDMS	7.7 × 10^9^	−68	−67.5	−63.9	0.404
S1050/PDMS	2.5 × 10^10^	−60	−59.2	−58.8	0.225
